# Supplementary material for: DiffractGPT: Atomic Structure Determination from X-ray Diffraction Patterns Using a Generative Pretrained Transformer
Source: J Phys Chem Lett. 2025 Feb 20;16(8):2110–9. doi: 10.1021/acs.jpclett.4c03137 (PMC11874033; doi:10.1021/acs.jpclett.4c03137)
Supplement: Supplementary file 1 — jz4c03137_si_001.pdf [file jz4c03137_si_001.pdf]

Supplementary information: DiffractGPT: Atomic Structure Determination from X-ray Diffraction Patterns using Generative Pre-trained Transformer

Kamal Choudhary*

Material Measurement Laboratory, National Institute of Standards and Technology, Gaithersburg, MD 20899, USA

E-mail: kamal.choudhary@nist.gov

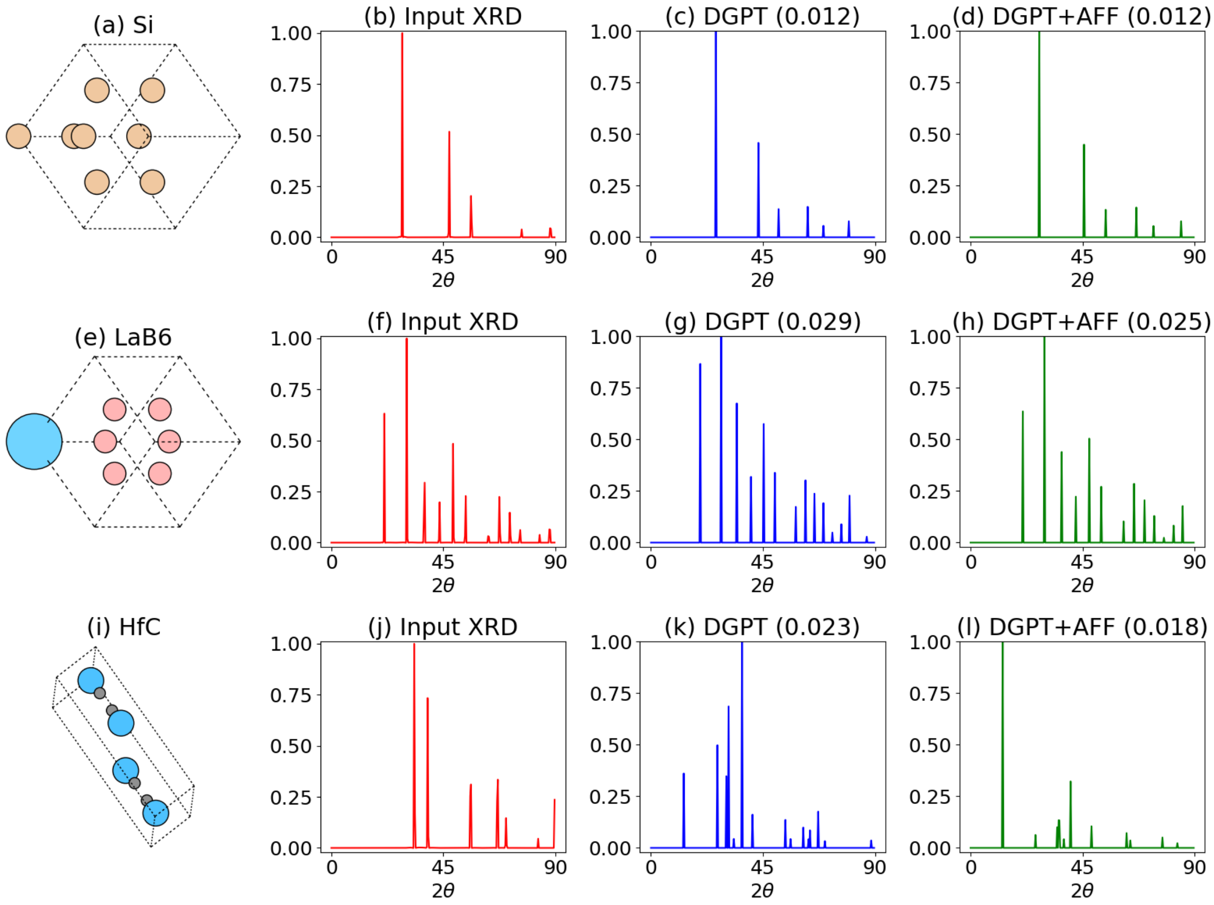

Figure S1: Evaluating the performance of DiffractGPT (DGPT)-formula model with and without ALIGNN-FF (AFF) optimization for a few example materials with experimental XRD patterns as inputs. The input chemical formula and XRD pattern are fed into the DGPT model to generate the atomic structure. The XRD pattern is scaled between 0 and 1 and peaks less than 0.04 as a threshold value are removed. The theoretical XRD pattern of the generated structure is shown as DGPT, along with the mean absolute error (MAE) of the XRD pattern in comparison with the input XRD. The DGPT structure is further optimized with AFF, and the XRD of the optimized structure, along with its MAE, is shown. (a) Silicon atomic structure, (b) input XRD pattern for Si, (c) XRD pattern of the DGPT-generated structure given the chemical formula and XRD, (d) XRD pattern for the AFF-optimized DGPT structure. Similar results for LaB_6 (e-h) and HfC (i-l) are shown.
